# Fungi of Tooth Caries

**Published:** 1883-04

**Authors:** W. D. Miller

**Affiliations:** Berlin


### FUNGI OF TOOTH CARIES.

BY DR. W. D. MILLER, BERLIN.

Translated from the Centralblatt of Medical Sciences

In the microscopical section of the Physiological Institute, investigations have been made by me on the fungi of tooth caries, which bring me to the following results:

1. The acids which are generated in the mouth through fermentation, withdraw the lime salts from the teeth.
2. In the tissues of teeth from which the lime salts have been withdrawn by the action of acids, a prolific growth of fungi *(Spaltpilze)* is found.
3. Leptothrix is found, with rare exceptions, only on the surface and in the superficial layers of the tissue; Bacili *(Stäbchen)* penetrate deeper, and micrococci furthest of all.
4. In the tooth tubuli we often find an undoubted gradual transition of the long bacteria into shorter ones, and these again into micrococci.
5. The invasion of fungi is always preceded by the action of acids.
6. The fungi are not able to withdraw the lime salts from the teeth, and a true infection of a thoroughly healthy tooth by one that is carious, is therefore impossible.
7. The fungi cause decay of the external portions, and pathological changes of the internal layers of the living tissue.

I reserve the right to report more fully on the intended experiments.

